# COVID-19 Pandemic Optimism and Vaccine Willingness among an Online Sample of US Gay, Bisexual, and Other Men Who Have Sex with Men

**DOI:** 10.3390/vaccines9070745

**Published:** 2021-07-05

**Authors:** Rob Stephenson, Stephen P. Sullivan, Renee A. Pitter, Alexis S. Hunter, Tanaka MD Chavanduka

**Affiliations:** 1 The Center for Sexuality and Health Disparities, Department of Systems, Populations and Leadership, School of Nursing, University of Michigan, Ann Arbor, MI 48109, USA; 2 The Center for Sexuality and Health Disparities, University of Michigan, Ann Arbor, MI 48109, USA; ssulliv@umich.edu (S.P.S.); raltrine@umich.edu (R.A.P.); ahun@umich.edu (A.S.H.); tanakac@umich.edu (T.M.C.)

**Keywords:** COVID-19, gay and bisexual men, vaccine

## Abstract

This paper presents data from an online sample of U.S gay, bisexual, and other men who have sex with men (GBMSM), to explore the factors associated with three dimensions of vaccine beliefs: perception of the likelihood of a COVID-19 vaccine becoming available, perception of when a COVID-19 vaccine would become available, and the likelihood of taking a COVID-19 vaccine. Data are taken from the Love and Sex in the Time of COVID-19 study, collected from November 2020 to January 2021. A sample of 290 GBMSM is analyzed, modeling three binary outcomes: belief that there will be a COVID-19 vaccine, belief that the COVID-19 vaccine will be available in 6 months, and being very likely to take the COVID-19 vaccine. In contrast to other studies, Black/African Americans and GBMSM living with HIV had higher levels of pandemic optimism and were more likely to be willing to accept a vaccine. Men who perceived a higher prevalence of COVID-19 among their friends and sex partners, and those who had reduced their sex partners, were more likely to be willing to take a COVID-19 vaccine. There remained a small percentage of participants (14%) who did not think the pandemic would end, that there would not be a vaccine and were unlikely to take a vaccine. To reach the levels of vaccination necessary to control the pandemic, it is imperative to understand the characteristics of those experiencing vaccine hesitancy and then tailor public health messages to their unique set of barriers and motivations.

## 1. Introduction

Since the initial case of COVID-19 was identified in the U.S in March 2020, COVID-19 has spread to all 50 States, and by April 2021, there were 30,737,471 confirmed cases and 556,106 deaths, the largest number of COVID-19 deaths in any country [1]. The primary response to the epidemic has been stay-at-home orders, which restrict social mobility and physical contact as a mechanism for limiting the spread of COVID-19 infection. These stay-at-home orders—rolled out at varying time points since March 2020 across the U.S—mean that approximately 316 million Americans have been urged to stay home [2]. As states moved in and out of lockdowns, there were resurgences, or ‘waves’ of COVID-19 cases in many states in the South (particularly Arizona, Florida, and Texas), in California, and in the Midwest (Michigan and Wisconsin) [3].

While the behavioral response to controlling the spread of COVID-19 has focused on limiting social contact, the biomedical response has focused on the development of vaccines. Since the genetic sequence for COVID-19 was published in early 2020 [4], the scientific community has raced to test, develop, and roll-out safe and effective COVID-19 vaccines. Although in early 2020, the World Health Organization (WHO) said it did not expect a COVID-19 vaccine to become available in less than 18 months [5], the rapid spread of the pandemic stimulated international alliances and government efforts to urgently organize resources to make multiple vaccines on shortened timelines [6]. In the U.S, three COVID-19 vaccines have been approved for use: Pfizer-BioNTech COVID-19 Vaccine (approved December 2020), Moderna COVID-19 Vaccine (approved December 2020), and the Janssen COVID-19 Vaccine (approved February 2021) [7]. By April 2021, 171 million vaccines had been administered to U.S adults [8].

Central to achieving the high levels of vaccination coverage needed to effectively control the spread of COVID-19 is overcoming vaccine hesitancy or the unwillingness among some to receive the vaccine. Since COVID-19 vaccines began to be developed in mid-2020, there has been an increase in studies examining vaccine willingness in multiple countries and populations. Daly et al. (2020) showed in a longitudinal sample of 7547 U.S adults that willingness to vaccinate against COVID-19 declined from 71% in April to 53.6% in October and was paralleled by an increase in the percentage of participants undecided about vaccinating (from 10.5% to 14.4%). In this sample, higher levels of vaccine unwillingness were identified among females, Black/African Americans, and those with lower levels of education [9]. Higher levels of willingness to vaccinate have been found among samples from several European countries (from 62% in France to approximately 80% in Denmark and the UK) [10], Australia (76.5% and 85%) [11,12,13], China (83.8%) [14], the UK (82%) [15], Japan (65.7%) [16] and the Democratic Republic of the Congo (55.9%) [17]. In a survey of 13,426 from 19 countries, Lazarus et al. (2021) [18] showed that vaccine acceptance rates ranged from 90% in China to 55% in Russia. Studies have found a number of factors commonly associated with the willingness to vaccinate against COVID-19 across samples. Higher levels of education [11,13,17,19], older age [12,16], those who perceive a higher level of seriousness of COVID-19 infection [12,20,21,22], levels of trust in science and vaccine development [20], and higher income levels [17,23,24] have all been associated with increased willingness to vaccinate against COVID-19. Several studies in the U.S and UK have also examined differences in vaccine willingness by race and ethnicity. Robertson et al. (2020) [15] used data from the UK Household Longitudinal Study to show that vaccine hesitancy was higher among Black (71.8%), South Asian (42.3%), and non-UK/Irish White (26.4%) groups. In an online survey of 2006 U.S adults, Reiter et al. (2020) [20] found that while 69% of participants were willing to take the COVID-19 vaccine, acceptance rates were significantly lower among Latinx and Black/African American participants. The lower levels of willingness to vaccinate against COVID-19 among communities of color have been attributed to high levels of medical mistrust in these communities [21] and the increased experience of structural vulnerabilities that limit access to health services.

While a number of studies have examined disparities in vaccine willingness by race and ethnicity, largely missing from the literature is an understanding of how vaccine willingness varies by sexual orientation. Sexual minority populations have experienced disproportionate impacts of the COVID-19 pandemic, with evidence of higher levels of economic impacts [25], poor mental health [25,26], and limited access to health services [25,26,27]. To date, only one study has examined willingness to receive COVID-19 vaccines among sexual minority groups. Using data from an online sample of 1350 U.S sexual and gender minority (SGM) individuals, Teixeira et al. (2021) [27] demonstrated that although vaccine acceptance was moderately high, measures of medical mistrust and COVID-19 vaccine stigma were significantly associated with decreased COVID-19 vaccine acceptance, while measures of altruism were significantly associated with increased vaccine acceptance. In their sample, Black/African American participants were significantly less likely to report willingness to accept a COVID-19 vaccine, and Asian participants were significantly more likely to accept a vaccine compared to White participants. Teixeira et al. (2021) [27] noted that a long history of stigma and discrimination aimed at SGM communities, particularly SGM communities of color, precipitates medical mistrust, with evidence that medical mistrust has been a barrier to the uptake of other vaccines in this community (i.e., HPV) [28] and in the use of general health care services [21,29].

In this paper we present unique data from an online sample of U.S gay, bisexual, and other men who have sex with men (GBMSM), to explore the factors associated with three dimensions of vaccine beliefs: perception of the likelihood a COVID-19 vaccine becoming available, perception of when a COVID-19 vaccine would become available, and the likelihood of taking a COVID-19 vaccine. We add to the literature by examining how these beliefs are associated with the perceived seriousness and perceived prevalence of COVID-19. Understanding the factors driving vaccine willingness in highly affected and stigmatized communities such as GBMSM has the potential to inform the targeting of vaccine promotion efforts to better recognize their unique needs and barriers.

## 2. Materials and Methods

Data for the current analysis were taken from the *Love and Sex in the Time of COVID-19* study, an online study conducted by the authors with U.S GBMSM, which consisted of 2 surveys, with the same participants surveyed in each round of data collection. The first round of data collection was conducted between April and May 2020; a 2nd round of data collection was conducted with the same participants from November 2020 to January, 2021. Participants were recruited online, using banner advertisements placed on two social networking platforms (Facebook and Instagram), and on a GBMSM specific app (Grindr). Advertisements were targeted to user profiles that were over the age of 18, identified as men, and currently reside in the U.S. The ads included the text, “*Still getting those DMs during quarantine? Take this survey about COVID-19 and sexual behaviors*”. Ethical approval for this study was obtained from the University of Michigan Institutional Review Board (IRB number HUM00180117).

Eligibility criteria were: (i) being over the age of 18, (ii) current resident of the U.S., (iii) assigned male sex at birth and currently identify as male, and (iv) reporting any type of sex in the past 12 months. Between April and May 2020, 34,930 participants clicked on the ads, 3864 people entered the survey portal, and 1789 (46.3%) started the survey. The primary reasons for ineligibility were: 11 (0.6%) reported living outside the U.S., 136 reported a gender other than male (7.6%), 5 reported being younger than 18 years of age (0.3%), and 283 had not had sex with a man in the past 12 months (15.8%). This resulted in 1354 eligible participants. In total, 696 (51.4%) participants completed the survey, for a final sample size of 696 GBMSM.

The 2nd round of data collection took place from November 2020 to January 2021, over a period of approximately 8 weeks, and consisted of a repeat survey with the same participants who completed round 1. The survey completion window was extended to account for anticipated slower rates of survey completion over the holiday season. Of the 696 GBMSM who completed round 1, 695 (99.9%) were emailed survey links (1 email bounced back). Of these, 348 (50.1%) completed round 2 of the survey. The demographic and behavioral profiles of participants in rounds 1 and 2 of the survey were similar, with 1 exception: a higher percentage of round 2 participants reported being employed (85.7%) compared to participants in round 1 (79.0%) (*p*-value 0.045) (data not shown).

The survey collected data on participant demographics, including self-reported age, race and ethnicity, employment status, educational attainment, sexual orientation, relationship status, and recent experience of indicators of incarceration and homelessness. The survey asked participants about their experience of COVID-19, including loss or reduction in employment and experiences of housing instability or food insecurity due to COVID-19. The ASSIST [30] and AUDIT [31] measures were used to measure the use of non-prescription drugs and alcohol: participants were also asked whether they felt their substance use or binge drinking (episodes of more than 5 alcoholic drinks) had increased during the COVID-19 lockdown. The survey collected data on the recent experience of COVID-19 testing and test results among those who reported that they had been tested in the past 3 months. Participants were asked to report the perceived prevalence of COVID-19 among the U.S. as a whole, in the participant’s state and among their friends, all measured on scales from 0–100. 

A scale measured the perceived seriousness of contracting COVID-19, consisting of 13 items that crossed 3 domains: personal (i.e., contracting COVID-19 would be very serious to me), psychosocial (i.e., contracting COVID-19 would jeopardize my relationships with my family), and physical (i.e., thinking about contracting COVID-19 stops me from sleeping). The scale had a potential range of 13 to 65, with higher scores indicating more perceived severity of COVID-19 infection.

Round 2 of the survey included questions on the perception of how long the COVID-19 pandemic would last, the likelihood of a vaccine, and their willingness to take a vaccine. Participants were asked “Do you think there will be an end to the COVID-19 pandemic?”, and those responding yes were asked, “When do you think the COVID-19 pandemic will end?” with options: in the next 6 months, 6–12 months, 1–3 years, 3–5 years, and more than 5 years. Participants were asked “Do you think there will be a vaccine for COVID-19?” (yes/no) and those responding yes were asked “When do you think there will be a vaccine for COVID-19?” (in the next 6 months, 6–12 months, 1–3 years, 3–5 years, and more than 5 years). All participants were asked: “How likely are you to take a vaccine for COVID-19 if it were available?” (very likely, somewhat likely, somewhat unlikely, and very unlikely).

Analysis used data from round 2 of the survey only. Of the 348 survey responses, 58 participants had missing data on the perception of the end of COVID-19 and the availability of a vaccine. There were no differences in demographic (i.e., age, race, education, or employment), behavioral (i.e., substance and alcohol use), or COVID-19 (participation in social distancing or COVID-19 testing) between those with and without missing data for these variables. The final analysis sample was 294 GBMSM.

The analysis focused on 3 binary outcomes. The first was coded 1 of if the participant reported that they believed there would be a COVID-19 vaccine. The 2nd was coded 1 if the participant reported that they believed there will be a COVID-19 vaccine in the next 6 months (those who reported they did not believe there will be a vaccine were coded as zero). The 3rd was coded 1 if the participant reported that they were very likely to take the COVID-19 vaccine. A binary variable was modeled for the likelihood of vaccine uptake as 78.1% of the sample reported they would be very likely to take the vaccine. Logistic models were fit for each of the 3 outcomes, each model including demographic characteristics, structural vulnerabilities (incarceration and homelessness), changes during the COVID-19 pandemic, and perceptions of the prevalence of COVID-19. All variables were entered into the models simultaneously. Analysis was completed in STATA version 16.0.

## 3. Results

The majority of the sample was aged between 25–44 (45.2%), was predominantly White (80.6%), gay identifying (85.0%), had some college education (43.9%), reported being HIV-negative (85.4%), and reported being currently employed (86.1%) (see Table 1). In terms of COVID-19 related influences, 22.8% of participants reported that their substance use had increased during the pandemic, and 34.4% reported that binge drinking had increased. Over half the sample (58.8%) reported that they had reduced their number of sex partners during the pandemic. Approximately one-third (35.7%) reported a loss of employment, and 7.3% reported difficulties with housing due to the COVID-19 pandemic. While almost all participants (98%) reported practicing social distancing, 14% reported having tested for COVID-19 in the past 6 months.

Participants believed that the prevalence of COVID-19 was higher among more distal groups, reporting a mean prevalence of 17.6% for the U.S, 18.7% for their state, and 18.8% for their local county. However, participants reported lower perceived COVID-19 prevalence in more proximal groups, with a mean prevalence of 13.6% among their friends and 12.5% among their sex partners.

The vast majority of participants felt that there would be an end to the COVID-19 pandemic (86.0%), but only 5.1% felt the end would be in the next 6 months (Table 2). However, 14% reported that they felt the pandemic would never end. The majority of participants felt the end of the pandemic would be in 6–12 months (42.9%). Almost all participants felt there would be a vaccine for COVID-19 (96.3%), and 46.5% felt the vaccine would be available in the next 6 months. Approximately three-quarters (78.1%) of participants reported they were very likely to take the vaccine, while only 3.2% said they were somewhat unlikely and 2.1% that they were very unlikely.

Table 3 presents the results of the modeling of beliefs that there will be a COVID-19 vaccine, that the vaccine will be available in the next 6 months, and being highly likely to take the vaccine. Older participants had significantly greater odds of reporting that they believed there would be a vaccine relative to those aged 18–24, but the belief that the vaccine would be ready within the next 6 months declined significantly with age. Higher levels of education were associated with significantly higher odds of believing there would be a vaccine and of being highly likely to take the vaccine. Black/African American participants and those who self-reported living with HIV were significantly more likely to report that there would be a vaccine, that it would be available in the next 6 months, and that they would be highly likely to take the vaccine. Those who perceived COVID-19 infection to be more serious were also more likely to report that there would be a vaccine, that it would be available in the next 6 months, and that they would be highly likely to take the vaccine.

Men who reported that they had tested for COVID-19 had significantly greater odds of reporting there would be a vaccine and that it would be available in the next 6 months but did not have significantly higher odds of saying they would be more likely to take the vaccine. Men who reported that they had reduced their number of sex partners during the pandemic also had significantly greater odds of believing there would be a vaccine and that they were highly likely to take the vaccine. Men who reported experiencing loss of employment due to COVID-19 had significantly lower odds of reporting there would be a vaccine, and men who reported that their binge drinking had increased during the pandemic had significantly higher odds of being very likely to take the vaccine.

Men who reported that the pandemic would not end for more than 12 months, or who felt the pandemic would never end, were less likely to believe there would be a vaccine, to think a vaccine would be available in the next 6 months, and be highly likely to take the vaccine. Men who felt that the prevalence of COVID-19 was higher in the U.S, their state, and their county were more likely to believe there would be a vaccine. Men who reported higher perceived prevalence of COVID-19 among their friends were more likely to believe a vaccine would be available in the next 6 months, and men who perceived higher COVID-19 prevalence among their friends and sex partners were more likely to be very likely to take the vaccine.

Relative to men in the North, men in the South were significantly less likely to believe there would be a vaccine and to be highly likely to take the vaccine, and men in the West were significantly more likely to report that the vaccine would be available in the next 6 months.

## 4. Discussion

The results demonstrate high levels of vaccine optimism and willingness among this online sample of GBMSM, paralleled by high levels of optimism for the end of the pandemic. Only a small percentage of men (14%) felt the pandemic would never end, and while 86% reported that although it would end, only 5% felt the pandemic would end in the next 6 months. Similarly, participants reported almost universal belief that there would be a vaccine (96.3%), however, there was more optimism that a vaccine would occur in the next 6 months (45.6%). GBMSM in this sample seems to anticipate that although a vaccine is imminent—the data were collected in November 2020 to January 2021, as vaccines were being approved for use—the availability of the vaccine may not translate into pandemic’s end, and that it will take a significant amount of time to roll out the vaccine to sufficient levels of uptake to impact the spread of COVID-19. However, willingness to take the vaccine was very high, at 78.1%, contrasting to the 53% willingness Daly et al. (2021) [9] reported for U.S adults in October 2020.

Older men were more likely to believe that there would be a vaccine but were less likely to believe the vaccine would be ready in the next 6 months, perhaps indicating a reduction in optimism with age. Those with higher levels of education were also more likely to believe there would be a vaccine and were more likely to be willing to take the vaccine, reflecting greater access to accurate information and greater social capital to access health care among those with higher levels of education. Men living with HIV were more accepting of COVID-19 vaccines: it is possible that HIV-positive men may have more concerns about the seriousness and perceived impact of COVID-19 infection on their health. Our results differ from previous studies in two important ways: Black/African American GBMSM and those living with HIV were more likely to believe there would be a vaccination, that it would be available in the next 6 months, and that they would take the vaccine. Teixeria et al. (2021) [27] showed that Black/African American SGM were less willing to accept a vaccine, and Bogart et al. (2021) [21] noted high levels of medical mistrust among HIV-positive African Americans. Several other recent studies have shown Black/African American participants to be less willing to take the COVID-19 vaccine [20,32,33,34].

It is possible that attitudes to the COVID-19 vaccine are influenced by time and ongoing socio-political events. The data collected by Teixeria et al. (2021) [27] were collected in October to December 2020, while ours were collected from November 2020 to January 2021. Vaccines began to be approved in the U.S in December 2020: hence the data from Teixeria et al. (2021) [27] were collected primarily pre-approval, while ours was collected during and immediately after approval. It is possible that the vaccine approval process influenced perceptions of vaccines, and GBMSM of color and those living with HIV who have been particularly impacted by the COVID-19 pandemic, are reporting greater willingness to be vaccinated in reaction to their perceived and actual disproportionately negative experience of the pandemic. Pre-FDA approval, vaccine perceptions centered on a vaccine that was on its way: however, once approved, and people had time to observe the roll-out process and the low levels of adverse events, it is possible that attitudes changed. Our data were collected after the election of U.S President Biden, and it is possible that this created a renewed sense of optimism and empowerment among African American/Black communities. Our survey did not measure medical mistrust, and so we are unable to examine the moderating effect that mistrust has on vaccine willingness and optimism among GBMSM of color, as was the central argument of Teixeria et al. (2021) [27]. However, our sample reported high levels of negative effects of the pandemic—including increases in substance use, binge drinking, loss of employment, and difficulties with housing. These negative experiences, along with the reported high levels of the perception of the seriousness of COVID-19 infection, are likely driving the high levels of willingness to be vaccinated.

Those who reported that they had reduced their number of sex partners and felt that COVID-19 was more serious were more likely to accept a COVID-19 vaccination, confirming recent studies that have also shown that those who are more concerned about COVID-19 are more likely to be accepting of vaccines [12,20]. It is possible that GBMSM may accept the vaccine as a strategy to allow them to return to pre-COVID-19 levels of sexual activity, although further work is warranted to understand this association. Reporting a higher perceived prevalence of COVID-19 at more proximal levels was associated with an increased willingness to accept a COVID-19 vaccine. Perceiving a higher prevalence among those around you likely indicates a higher perceived risk of infection, which is translating to an increased willingness to be vaccinated.

The small percentage of GBMSM who reported that the pandemic would not end had the lowest odds of believing there would a vaccine, a vaccine would be available in the next 6 months, and that they would take a vaccine. Rothmund et al. (2020) [35] reported that deniers (who comprised 8% of their sample) are characterized by low-risk assessments and low compliance with containment measures, while doubters (who comprised 19% of their sample) were characterized by general uncertainty in the distinction between true and false claims and by low scientific literacy. The current survey did not measure attitudes and beliefs about the accuracy of the pandemic, or general beliefs or trust in science, and so it is not possible to accurately refer to this group of participants as COVID-19 deniers or doubters. However, there is clearly a need to both understand the demographic and behavioral profiles of GBMSM who report COVID-19 pessimism and anti-vaccine beliefs and to develop tailored public health messaging that address and correct these beliefs.

There are several limitations to the current study. The data were collected online, and, therefore, represent only those with access to the internet. The survey was conducted from November 2020 to January 2021, just as vaccines were emerging, and GBMSM may have been in the early stages of forming their opinions of the vaccines or waiting for more information before making a decision on vaccination. It would be important to repeat this survey later in 2021, to understand whether vaccine attitudes and pandemic optimism have changed further. The sample size was relatively small, and further work with larger, more diverse samples is necessary. To be eligible for the study, men needed to report having sex in the past 12 months. It is possible that those who were not having sex and were thus excluded from the study may have different attitudes towards COVID-19 vaccines. The survey did not collect measures of medical mistrust or measures of trust or belief in science, limiting some of the conclusions. The sample is predominantly White and highly educated. With a more racially and economically diverse sample, we may expect to observe greater variation in the negative experiences of COVID-19.

## 5. Conclusions

The results illustrate high levels of pandemic optimism and vaccine willingness among an online sample of GBMSM who have experienced significant negative impacts of the COVID-19 pandemic. The results show for the first time that GBMSM of color and those living with HIV are more willing to accept a COVID-19 vaccine and have higher levels of vaccine optimism, which we suggest indicates changes in beliefs as vaccines are approved and become available. There remains, however, a small percentage of GBMSM who do not think the pandemic will end and are unwilling to accept a vaccine. The results have several implications for public health strategies that aim to improve the uptake of COVID-19 vaccinations. Identifying the characteristics of those with these levels of vaccine willingness and pandemic optimism can highlight messaging strategies to be used in vaccine promotion efforts. For example, results showing the associations between the perceived prevalence of COVID-19 and willingness to be vaccinated indicate that messages including information on local COVID-19 prevalence may be a useful strategy for shifting opinions towards vaccine acceptance. To reach the levels of vaccination necessary to control the pandemic, it is imperative to focus on those with negative beliefs, and further qualitative work is warranted to understand the factors driving these beliefs. Such work, coupled with the results presented here, is a critical step in developing public health messages that target the unique barriers to vaccine uptake experienced by GBMSM.

## Figures and Tables

**Table 1:** Demographic and behavioral characteristics of an online sample of gay, bisexual, and other men who have sex with men (GBMSM) (*n* = 294).

Characteristic	%(n)
**Age** 18–24 25–34 35–44 >45 **Education** High school Some college College graduate or graduate school **Employed** Yes No **Race** Black/African American/Other White **Sexual identity** Gay/homosexual Bisexual/Other **HIV sero-status** HIV-negative/unknown HIV-positive **Changes in substance use during lockdown** Increased Decreased/Stayed the same **Changes in binge drinking during lockdown** Increased Decreased/Stayed the same **Reduced sex partners due to COVID-19** Yes No **Experienced loss of employment due to COVID-19** No Yes **Have experienced difficulties with housing due to COVID-19** No Yes Perceived seriousness of COVID-19 infection **Tested for COVID-19** No Yes **Practiced social distancing** No Yes Perceived prevalence of COVID-19 among US population Perceived prevalence of COVID-19 among state population Perceived prevalence of COVID-19 among county population Perceived prevalence of COVID-19 among friends Perceived prevalence of COVID-19 among sex partners **Region** North East South West	15.3 (45) 45.2 (133) 27.9 (82) 11.6 (34) 21.1 (62) 43.9 (129) 35.0 (103) 86.1 (253) 13.9 (41) 19.4 (57) 80.6 (237) 85.0 (250) 15.0 (44) 85.4 (521) 14.6 (43) 22.5 (66) 77.5 (228) 34.4 (101) 65.6 (193) 58.8 (173) 41.2 (121) 64.3 (189) 35.7 (105) 92.7 (273) 7.3 (21) 33.4 (−81–60) 86.0 (253) 14.0 (41) 2.0 (6) 98.0 (288) 17.6 (0–91) 18.7 (0–90) 18.8 (0–93) 13.6 (0–80) 12.5 (0–100) 16.0 (47) 29.6 (87) 23.5 (69) 30.9 (91)

**Table 2:** Attitudes towards the COVID-19 pandemic and the future of a COVID-19 vaccine in an online sample of gay, bisexual, and other men who have sex with men (GBMSM) (*n* = 294).

	*n* (%)
**The COVID-19 pandemic will end** Yes No **When will the COVID-19 pandemic end** Within the next 6 months Within the next 6 months to 1 year In 1 to 3 years In 3 to 5 years Never **There will be a vaccine for COVID-19** Yes No **When will there be a vaccine for COVID-19** Within the next 6 months Within the next 6 months to 1 year In 1 to 3 years In 3 to 5 years Never **How likely are you to take the COVID-19 vaccine** Very likely Somewhat likley Somewhat unlikely Very unlikley	86.0 (253) 14.0 (41) 5.1 (15) 42.9 (126) 37.8 (111) 0.3 (1) 14.0 (41) 96.3 (283) 3.7 (11) 46.5 (132) 41.1 (116) 12.1 (34) 0.3 (1) 3.7 (11) 78.1 (221) 16.6 (47) 3.2 (9) 2.1 (6)

**Table 3:** Logistic regression models for self-reported beliefs in availability and timing of a COVID-19 vaccine and likelihood of taking a COVID-19 vaccine in an online sample of gay, bisexual and other men who have sex with men (GBMSM) (*n* = 294).

	Believes There Will Be a COVID-19 Vaccine	Believes There Will Be a COVID-19 Vaccine within 6 Months	Reports Being Highly Likely to Take the COVID-19 Vaccination OR (95% CI)
Characteristic	OR (95% CI)	OR (95% CI)	OR (95% CI)
**Age** (18–24) 25–34 35–44 >45 **Education** (High school) Some college College graduate or graduate school **Employed** (Yes) No **Race** (White) Black/African American/other **Sexual identity** (Gay/homosexual) Bisexual/Other **HIV sero-status** (HIV-negative) HIV-positive **Substance has increased during lockdown** (No) Yes **Binge drinking has increased during lockdown (No)** Yes **Experienced loss of employment due to COVID-19 (No)** Yes **Have experienced difficulties with housing due to COVID-19 (No)** Yes **Reduced number of sex partners to avoid COVID-19 infection (No)** Yes Perceived seriousness of COVID-19 infection **Tested for COVID-19 (No)** Yes **Believes COVID-19 pandemic will end (in the next 12 months)** Yes, but more than 12 months No Perceived prevalence of COVID-19 among US population Perceived prevalence of COVID-19 among state population Perceived prevalence of COVID-19 among county population Perceived prevalence of COVID-19 among friends Perceived prevalence of COVID-19 among sex partners Region (North) East South West	*3.32 (0.45, 0.98)* *3.30 (0.37, 0.96)* *2.25 (0.16, 0.88)* *4.81 (0.17, 0.74)* *1.93 (0.29, 0.78)* 1.33 (0.28, 2.01) *1.86 (1.25, 2.41)* 0.41 (0.30, 1.21) *5.48 (2.21, 7.14)* 0.96 (0.50, 1.41) 1.59 (0.20, 2.36) *0.29 (0.10, 0.54)* 0.83 (0.51, 1.34) *1.62 (1.10, 2.51)* *1.10 (1.02, 1.22)* *1.35 (1.10, 1.52)* *0.89 (0.74, 0.96)* *0.54 (0.23, 0.76)* 1.10 (0.91, 1.17) 1.05 (0.91, 1.21) *1.24 (1.10, 1.34)* *1.10 (1.01, 1.21)* *1.12 (1.03, 1.22)* 0.69 (0.34, 1.05) *0.54 (0.23, 0.76)* 1.02 (0.98, 1.08)	*0.41 (0.18, 0.92)* *0.39 (0.16, 0.92)* *0.35 (0.12, 0.97)* *1.54 (0.76, 2.25)* *2.19 (0.99, 3.01)* *0.64 (0.29, 1.37)* *1.79 (1.21, 2.24)* *0.60 (0.29, 1.21)* *1.39 (1.15, 1.63)* *0.87 (0.45, 1.71)* *1.07 (0.59, 1.93)* *1.21 (0.70, 2.09)* *0.93 (0.34, 2.72)* *0.68 (0.40, 1.16)* *1.05 (1.01, 1.10)* *1.10 (1.01, 1.25)* *0.18 (0.10, 0.33)* *0.15 (0.06, 0.35)* *1.01 (0.97, 1.13)* *1.07 (0.97, 1.14)* *0.98 (0.95, 1.02)* *1.10 (1.02, 1.23)* *0.99 (0.94, 1.02)* *1.82 (0.77, 2.89)* *1.12 (0.45, 2.80)* *2.74 (1.14, 3.59)*	0.60 (0.23, 1.53) 0.60 (0.21, 1.68) 0.65 (0.19, 2.18) 1.31 (0.58, 2.18) *3.17 (1.20, 5.40)* 0.73 (0.31, 1.72) *1.76 (1.35, 2.24)* 1.58 (0.69, 2.60) *1.15 (1.04, 1.31)* 1.01 (0.44, 2.17) *1.91 (1.44, 2.53)* 1.14 (0.54, 2.01) *1.37 (1.03, 1.67)* *1.71 (1.36, 2.64)* *1.15 (1.04, 1.29)* 0.84 (0.41, 1.69) *0.76 (0.28, 0.98)* *0.11 (0.04, 0.25)* 1.01 (0.97, 1.04) 0.97 (0.93, 1.01) 1.04 (0.97, 1.03) *1.12 (1.02, 1.27)* *1.13 (1.04, 1.23)* 1.48 (0.57, 3.74) *0.74 (0.48, 0.96)* 1.37 (0.43, 3.50)

Figures in italics are significant at the 5% level.

## Data Availability

The data presented in this study are available on request from the corresponding author. The data are not publicly available due to privacy and ethical restrictions.
